# Asymmetrical Interference Effects between Two-Dimensional Geometric Shapes and Their Corresponding Shape Words

**DOI:** 10.1371/journal.pone.0092740

**Published:** 2014-03-20

**Authors:** Bradley R. Sturz, Joshua E. Edwards, Ty W. Boyer

**Affiliations:** Department of Psychology, Georgia Southern University, Statesboro, Georgia, United States of America; University of Akron, United States of America

## Abstract

Nativists have postulated fundamental geometric knowledge that predates linguistic and symbolic thought. Central to these claims is the proposal for an isolated cognitive system dedicated to processing geometric information. Testing such hypotheses presents challenges due to difficulties in eliminating the combination of geometric and non-geometric information through language. We present evidence using a modified matching interference paradigm that an incongruent shape word interferes with identifying a two-dimensional geometric shape, but an incongruent two-dimensional geometric shape does not interfere with identifying a shape word. This asymmetry in interference effects between two-dimensional geometric shapes and their corresponding shape words suggests that shape words activate spatial representations of shapes but shapes do not activate linguistic representations of shape words. These results appear consistent with hypotheses concerning a cognitive system dedicated to processing geometric information isolated from linguistic processing and provide evidence consistent with hypotheses concerning knowledge of geometric properties of space that predates linguistic and symbolic thought.

## Introduction

For centuries nativists and empiricists have debated the extent to which spatial thinking is innate or learned [1]. Recent scientific inquiry has provided evidence in support of innate spatial mechanisms for navigation and orientation via geometric cues [2]–[7] as well as for facial recognition via spatial properties [8], and the strongest evidence in support of a nativist approach comes from cross-species experiments on geometric encoding of the environment [9]–[13], see also [14]–[15]. Experiments conducted on species from ants to adult humans have provided evidence that incidental learning of geometric properties of an environment is a fundamental and ubiquitous component of spatial cognition that occurs across phylogeny and ontogeny [9]–[10]. Such evidence has been used in support of hypotheses for Euclidean geometry as one of many domains of core knowledge that predate linguistic and symbolic thought [11]–[13], [16]–[17].

Central to these hypotheses is the proposal for an isolated modular cognitive system dedicated to processing geometric information [3], [6], [11]–[13], [18]–[19]. Tests of such hypotheses with normally functioning adults who display well-developed linguistic systems have presented numerous challenges, and one of the greatest challenges relates to the difficulty in eliminating the combination of geometric and non-geometric information (e.g., color) through the use of language [20], c.f., [21]–[22]. In short, an adult's ability to encode spatial relations linguistically (e.g., “the dog is in front of the tree”) precludes researchers' ability to investigate the use of pure geometric information isolated from linguistic processing. Although researchers have attempted to disrupt the encoding of spatial relations in a linguistic fashion through the implementation of distractor tasks [20]–[22], evidence for such a modular cognitive system isolated from linguistic processing remains elusive in normal functioning adults. As a result, any evidence of such an isolation of geometric processing from linguistic processing in adult participants with well-developed linguistic systems would assist in illuminating potential evolutionary and developmental origins of spatial and linguistic processes.

**Figure 1:**
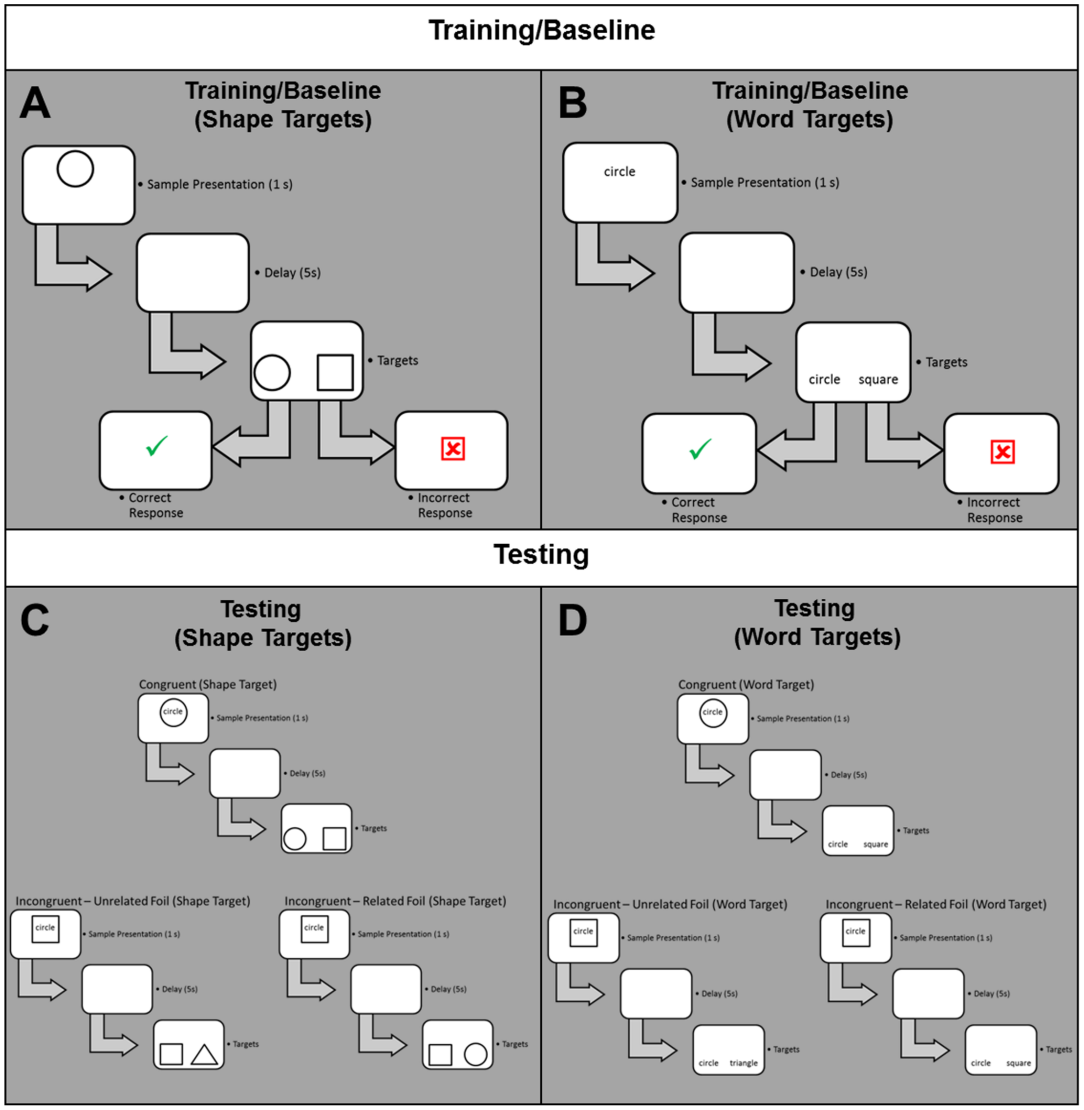
Sample trial types and trial structures for the Delayed Match-to-Sample (DMTS) task. One sample Baseline/Training trial is illustrated for Shape Targets (**A**) and Word Targets (**B**), and one sample Congruent, Incongruent – Unrelated Foil, and Incongruent – Related Foil trial is illustrated for Shape Targets (**C**) and Word Targets (**D**). For illustrative purposes, all correct matches are shown as the left target even though correct target and foil target locations were balanced (see text for details).

In the present experiment, we modified an interference paradigm to probe the isolation of geometric and linguistic processing [23]–[24]. By modifying a match-to-sample task that has previously provided evidence for semantic interference [24], we were able to present bi-dimensional samples composed of a two-dimensional geometric shape and a shape word (see Figure 1) and manipulate whether the shape and shape word were congruent (e.g., “circle” in a circle) or incongruent (e.g., “circle” in a square). After a delay, we probed each sample dimension independently during target presentation via shape targets (i.e., two shapes) or word targets (i.e., two words; Figure 1A/C and Figure 1B/D, respectively) and manipulated whether the incorrect response option was related or unrelated to the irrelevant sample dimension.

We believe this approach is uniquely suited to probe the isolation of geometric and linguistic processing because it allows for determination of the extent to which a two-dimensional geometric shape interferes with identification of a shape word as well as determination of the extent to which a shape word interferes with identification of a two-dimensional geometric shape. In the presence of a bi-dimensional stimulus composed of a two-dimensional geometric shape and a shape word that are incongruent (e.g., “circle” in a square), the sample word dimension could activate a spatial representation of the shape, and this spatial representation could interfere with identifying the sample shape dimension in the presence of two shape targets. Reciprocally, the sample shape dimension could activate a linguistic representation of the shape, and this linguistic representation could interfere with identifying the sample word dimension in the presence of two word targets.

From a strict empiricist perspective, interference effects should be symmetrical for shape and word targets such that RTs on trials in which a shape and shape word are presented in isolation (i.e., Baseline trials, see Figure 1A/B) or are congruent (i.e., Congruent trials, see Figure 1C/D) should not differ, but both of these trial types should be faster than trials in which the shape and shape word are incongruent (i.e., Incongruent – Unrelated Foil and Incongruent – Related Foil trials, see Figure 1C/D). Importantly, these trial type RT effects should hold for *both* shape and word targets, and interference by the irrelevant sample dimension should be reflected in accuracy measures of *both* shape and word targets – especially in the presence of an incorrect response option that is related to the irrelevant sample dimension (due to a greater probability of error in the presence of two potential matches on the basis of linguistic or spatial representations of the sample dimensions).

In contrast, under the assumption of a modular cognitive system dedicated to processing geometric information isolated from linguistic processing [11]–[13], [16]–[18], interference effects should be asymmetrical. A shape should not activate a linguistic representation (i.e., a circle should not activate the word “circle”). As a result, an incongruent shape should not interfere with identifying the relevant sample word dimension in the presence of two word targets. To the extent that geometric processing is isolated from linguistic processing, the predictions outlined above regarding the trial type RT and accuracy effects should hold for shape targets but not word targets.

## Methods

### Participants

Twenty-four undergraduate students at Georgia Southern University (12 males; 12 females) served as participants. Participants had normal or corrected-to-normal vision and received extra class credit or participated as part of a course requirement.

### Ethics statement

The research was conducted following the relevant ethical guidelines for human research. We obtained written informed consent from all participants, and all procedures were approved by Georgia Southern University's Institutional Review Board.

### Apparatus

We constructed and implemented a delayed match-to-sample task (see Figure 1) on a personal computer with a 22-inch flat-screen liquid crystal display (LCD) monitor (1,680×1,050 pixels). Responses occurred via the “c” (left target) and “m” (right target) keys on a standard keyboard. Experimental events were controlled and recorded using E-Prime (Psychology Software Tools, Inc., www.pstnet.com).

### Stimuli

There were two stimulus types: Shapes (Figure 1 A/C) and Words (Figure 1 B/D). Shape stimuli were circles, squares, and triangles each presented in a 5 pixel width black outline measuring 312 pixels in diameter (circle), 312 pixels in height and width (square), and 440 pixels in base width and 312 pixels in height (triangle) subtending 7.3° visual angle horizontally and vertically (circle and square) and 10.3° horizontally and 7.3° vertically (triangle). Word stimuli were “circle”, “square”, and “triangle” presented in bold 40 point Courier New font and were 187 (“circle” and “square”) and 250 (“triangle”) pixels in width, subtending 4.4° (“circle” and “square”), and 5.9° (“triangle”) visual angle horizontally, and 34 (“circle” and “square”) or 44 (“triangle”) pixels in height, subtending 0.8° or 1.0° visual angle vertically. Words were presented in black font color. All stimuli were presented on a white background. Samples were presented in the horizontal center of the screen 25% down from its top edge. Targets were presented on opposite sides of the screen, 50% of screen width apart, and 25% up from its bottom edge.

### Procedure

We provided participants with instructions that they would complete a memory test in which one of several shapes and words would appear on the screen, would disappear, and then either a pair of shapes or words would appear. Instructions also informed them that their task would be to select the shape that matched the sample shape (if shape pairs) or select the word that matched the sample word (if word pairs).

The experimental protocol consisted of 120 total trials for each participant composed of 24 Training Trials and 96 Testing Trials. All trials presented samples for 1 s, followed by a 5 s blank screen retention interval delay, followed by target stimuli for 1.5 s. A response to the correct target (i.e., match) resulted in the presentation of a green check mark; a response to the incorrect target (i.e., foil) resulted in the presentation of a red “X”, and failure to respond during the 1.5 s target presentation produced a “No Response” statement. Feedback was presented for 1 s, and served as the inter-trial interval (ITI).

#### Training

To familiarize participants with the task, we provided them with 24 training trials composed of two 12-trial blocks. One block included 12 unique shape training trials in which participants matched a sample shape to its corresponding shape target (Figure 1A), and the other block included 12 unique word training trials in which participants matched a sample word to its corresponding word target (Figure 1B). We balanced for gender and counterbalanced the training blocks order of presentation.

#### Testing

Testing consisted of 96 trials composed of 12 eight-trial blocks. Each trial block was composed of two trials of each of four trial types (see Figure 1): Baseline (Training), Congruent (sample shape with corresponding shape word), Incongruent – Unrelated Foil (sample shape with non-corresponding shape word and a foil unrelated to the irrelevant sample dimension), and Incongruent – Related Foil (sample shape with non-corresponding shape word and a foil related to the irrelevant sample dimension). Baseline trials were identical to Training trials. For all trial types, when shape targets were presented (e.g., circle and square), the corresponding sample shape was the correct response. When words targets were presented (e.g., “circle” and “square”), the corresponding sample word was the correct response.

We presented one trial with shape targets and one trial with word targets for each trial type within each block in randomized sequences. The left/right location of the correct target (i.e., match) and foil were counterbalanced, which resulted in each unique combination of each trial type being presented once, without replacement, for a total of 96 trials during Testing (24 Baseline trials, 24 Congruent trials, 24 Incongruent – Unrelated Foil trials, and 24 Incongruent – Related Foil trials). Feedback was identical to Training.

## Results

We analyzed Testing data via RTs and proportions correct.

### Response time

**Figure 2:**
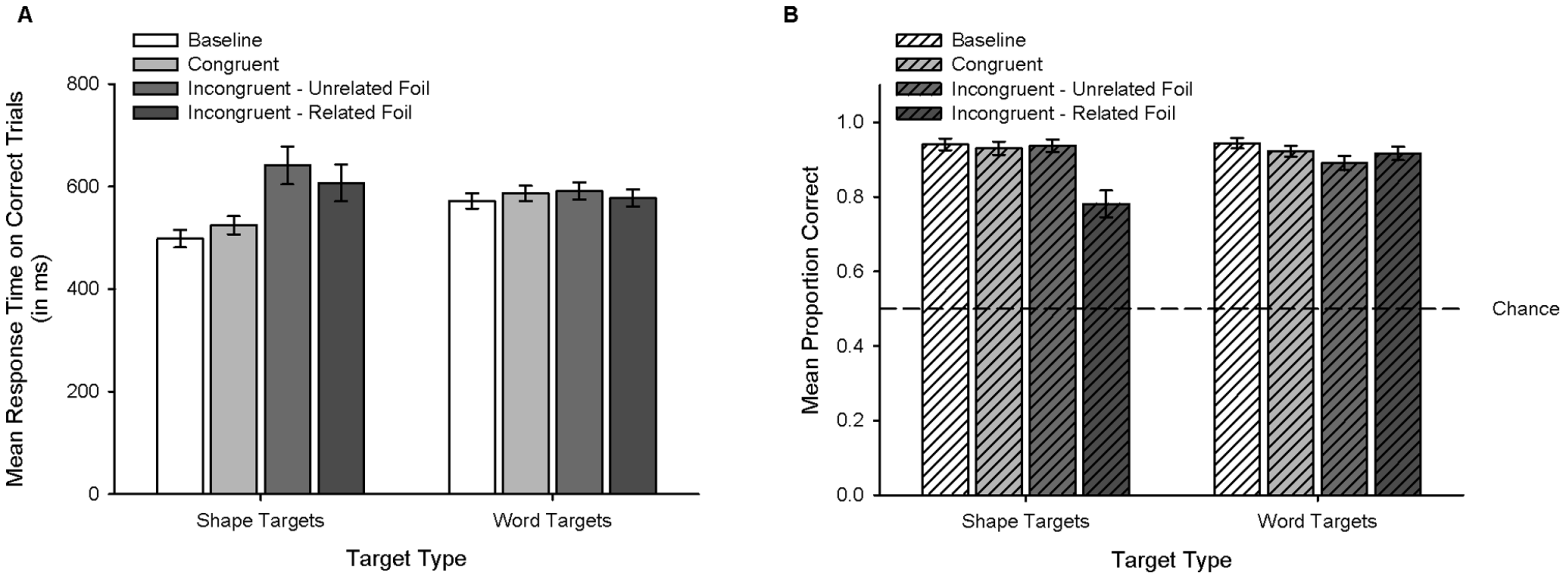
Performance During Testing. (**A**) Mean response time on correct trials during Testing (in milliseconds) plotted by Target Type for each Trial Type. (**B**). Mean proportion correct during Testing plotted by Target Type for each Trial Type. Dashed line represents chance performance (0.5). Error bars represent standard errors of the means.

We analyzed correct trials (error rates opposite of proportion correct shown Figure 2B). Figure 2A shows the mean RTs (in ms) plotted by Target Type for each Trial Type. A two-way repeated measures analysis of variance (ANOVA) on RT with Target Type (shape, word) and Trial Type (baseline, congruent, incongruent – unrelated foil, incongruent – related foil) as factors revealed a main effect of Trial Type *F*(3, 69)  = 11.47, *p*<.001, η*_p_* ^2^  = 0.33, but a non-significant effect of Target Type, *F*(1, 23)  = 0.77, *p* = .39. These results were qualified by a significant Target Type x Trial Type interaction, *F*(3, 69)  = 13.47, *p*<.001, η*_p_* ^2^  = 0.37. To illuminate the source of the interaction, we conducted two separate one-way repeated measures ANOVAs for each Target Type with Trial Type (baseline, congruent, incongruent – unrelated foil, incongruent – related foil) as a factor. For the Shape Targets, there was a main effect of Trial Type, *F*(3, 69)  = 15.30, *p*<.001, η*_p_* ^2^  = 0.40. Post hoc tests revealed that Baseline and Congruent trials were not significantly different from each other (*p* = .07), but both of these trial types were significantly faster than Incongruent – Unrelated Foil and Incongruent – Related Foil trials (*p*s <.01). Incongruent – Unrelated Foil and Incongruent – Related Foil trials were not significantly different from each other (*p* = .09). For Word Targets, the main effect of Trial Type was not significant, *F*(3, 69)  = 0.95, *p* = .42.

### Proportion correct

We eliminated trials in which participants failed to respond (30/2304; 1.3%). Figure 2B shows the mean proportion correct plotted by Target Type for each Trial Type. A two-way repeated measures ANOVA on proportion correct with Target Type (shape, word) and Trial Type (baseline, congruent, incongruent – unrelated foil, incongruent – related foil) as factors revealed a main effect of Trial Type, *F*(3, 69)  = 10.8, *p*<.001, η*_p_* ^2^  = 0.32, but a non-significant effect of Target Type, *F*(1, 23)  = 2.36, *p* = .14. These results were qualified by a significant Target Type x Trial Type interaction, *F*(3, 69)  = 8.79, *p*<.001, η*_p_* ^2^  = 0.28. To illuminate the source of the interaction, we conducted two separate one-way repeated measures ANOVAs for each Target Type with Trial Type (baseline, congruent, incongruent – unrelated foil, incongruent – related foil) as a factor. For the Shape Targets, there was a main effect of Trial Type, *F*(3, 69)  = 12.92, *p*<.001, η*_p_* ^2^  = 0.36. Post hoc tests revealed that Baseline, Congruent, and Incongruent – Unrelated Foil trials were not significantly different from each other (*p*s >.64), but all three of these trial types were significantly more accurate than Incongruent – Related Foil trials (*p*s <.01). For Word Targets, the main effect of Trial Type was not significant, *F*(3, 69)  = 2.38, *p* = .08. All mean proportions correct were significantly greater than chance (0.5), one-sample *t*-tests, *t*s(23) >7.8, *p*s <.001.

It should be noted that an analysis identical to that reported above including errors of omission yielded qualitatively identical statistical results. It should also be noted that we conducted a follow-up experiment with 24 new participants. This follow-up experiment substituted nonsense words for shape words and confirmed that the trial type differences for shape targets did not result from facilitation of redundant sample cues. Congruent trials did not differ from Incongruent trials in measures of RT or accuracy. These results provide converging evidence that an incongruent shape word interferes with the identification of a shape but an incongruent shape does not interfere with the identification of a shape word - providing further support for an interpretation that shape words activate spatial representations of shapes but shapes do not activate linguistic representations of shape words.

## Discussion

Although RTs did not differ across trial types for Word Targets, RTs for both incongruent trial types were significantly slower than Baseline and Congruent trials for Shape Targets. Accuracy analyses indicated a decrement in performance only for Shape Targets on trials in which the foil was related to the irrelevant sample dimension (i.e., Incongruent – Related Foil trials) suggesting that interference by the sample word dimension resulted in two potential relevant matches on the basis of spatial representations during target presentations. These results appear to be opposite of a speed-accuracy trade-off and corroborate an interpretation of shape words activating spatial representations of shapes, but shapes not activating linguistic representations of shape words.

Collectively, we provide evidence for an asymmetry in shape and shape word interference such that, in the presence of a bi-dimensional stimulus composed of a shape and shape word, an incongruent shape word interferes with identifying a shape, but an incongruent shape does not interfere with identifying a shape word. Although our inclusion of adult participants with well-developed linguistic systems prevents us from drawing definitive conclusions about the evolutionary and developmental origins of spatial and linguistic processes, these findings are consistent with recent evidence that shape recognition processes emerge before, interact with, and enable the development of linguistic shape categories [25]–[26]. We believe our results are also consistent with nativists approaches that would suppose asymmetrical effects for spatial and linguistic processes, and, by extension, our results appear to provide evidence consistent with hypotheses concerning core knowledge for geometric properties of space that predates linguistic and symbolic thought [11]–[13], [16]–[18]. In contrast, our results appear largely inconsistent with strict empiricist approaches that would suppose symmetrical effects for the emergence of spatial and linguistic processes. Future research should be able to utilize the current paradigm coupled with neural imaging techniques to isolate associated brain regions and further substantiate our behavior results while providing converging evidence for a cognitive system dedicated to processing geometric information isolated from linguistic processing.
